# Supplementary material for: Inequalities in financial burden of tuberculosis among affected families across 19 low- and middle-income countries
Source: PLOS Glob Public Health. 2026 Apr 3;6(4):e0006172. doi: 10.1371/journal.pgph.0006172 (PMC13048425; doi:10.1371/journal.pgph.0006172)
Supplement: Attachment — Submitted filename: Response to Reviewers.docx [file pgph.0006172.s002.docx]

The Editor

PLOS Global Public Health

12^th^ February 2026

Ref: PGPH-D-25-02325

Title: INEQUALITIES IN THE FINANCIAL BURDEN OF TUBERCULOSIS AMONG AFFECTED FAMILIES: EVIDENCE FROM 19 LOW- AND MIDDLE-INCOME COUNTRIES

Response to Reviewers' Comments

Dear Sir/Madam,

We want to express our sincere thanks for painstakingly reviewing our manuscript and providing valuable comments and suggestions. Please see our point-by-point response to the reviewers' comments and suggestions. Revisions are highlighted with track changes in the revised manuscript.

**Reviewer 1**

**Comment 1:** This manuscript describes an analysis of TB patient cost surveys using WHOs publicly available Health Equity Assessment Tool. The paper is clearly written and highlights the disparity in catastrophic costs between individuals with drug-susceptible and drug-resistant TB. It also highlights differences between countries. I do not have any major comments on the paper.

Response: Thank you

**Comment 2:** I have one minor question about the survey data. While these were carried out using a standardized WHO tool they were done in different years (2015-2020). Were there any changes to the WHO tool over this period? Or could differences in time period explain some of the between country differences observed e.g. changes in DR-TB regimens used?

Response: Thank you for your insightful feedback regarding the survey data collected using the WHO TB Patient Cost Survey tool across different years (2015-2020). We have reviewed the WHO documentation and confirmed that while minor updates to the tool occurred during this period, they did not affect the core indicators related to financial burden, ensuring data comparability. We also acknowledge that temporal differences, such as changes in DR-TB regimens or economic conditions, could contribute to between-country variations. To address this, we have added a brief paragraph in the Discussion section under "Limitations" to discuss these factors. We hope this clarifies the concern, and we remain open to further suggestions.

**Reviewer #2:**

**Comment 1:** This manuscript presents a cross-country analysis of catastrophic costs faced by tuberculosis-affected households across 19 low- and middle-income countries, using national TB Patient Cost Surveys and the WHO Health Equity Assessment Toolkit. The authors focus on inequalities by drug resistance status, comparing drug-susceptible TB and drug-resistant TB using multiple inequality measures (Difference, Ratio, Population Attributable Risk, and Population Attributable Fraction). The study highlights substantial financial burden and marked disparities, with drug-resistant TB–affected families consistently experiencing higher catastrophic costs.

**Overall Assessment**

**Comment 1:** Overall, this is a timely and policy-relevant manuscript that provides a valuable multi-country assessment of inequalities in catastrophic TB-related costs, with a particular focus on differences between DS-TB and DR-TB-affected households. The analysis is generally well conducted and clearly presented, and the use of multiple absolute and relative inequality measures strengthens its contribution to the TB equity literature. However, several issues related to conceptual framing, methodological transparency (particularly regarding the use of WHO HEAT), and interpretation of inequality measures would benefit from clarification to improve rigor, reproducibility, and policy relevance prior to publication.

Response: Thanks for highlighting these critical omission. We have attempted to address all the concerns raised.

**Comment 2:** Furthermore, in my opinion the overall analysis remains somewhat descriptive and would benefit from greater analytical depth. In its current form, the study largely relies on pre-loaded data and automated inequality measures within the WHO HEAT software, with limited examination/questioning of the assumptions behind the analysis, how reliable the results are, or what factors (in country eg TB/healthcare financing & drug availability, programmatic) might explain the differences seen. The study would also benefit from a more thorough explanation/justification for inequality measures and what other measures might be available (for benchmarking), or at least their limitations.

Response: We acknowledge the importance of conducting in-depth inferential analysis beyond the descriptive findings presented. However, we would like to clarify that the WHO HEAT toolkit does not contain disaggregated data to enable inferential analysis, making it impossible to perform such analysis at this time. To enhance transparency, we have added text in the Methods section of the abstract and the Discussion section under study limitations, explicitly stating that no inferential statistics were conducted.

Regarding the justification of inequality measures, we have expanded the explanation in the Outcome Measures subsection of the Methods section to provide a thorough rationale for selecting drug resistance TB type (with subgroups Drug Susceptible TB [DS-TB] and Drug Resistant TB [DR-TB]) as the dimension of inequality. Additionally, we have discussed alternative measures and their limitations to provide context and benchmarking. We believe these revisions address your concerns and improve the clarity of our methodology.

**Comment 3:** Line 20: Abstract introduction: “majority” of cases

Response: Added.

Introduction

**Comment 4:** The burden of TB is well described. The early paragraphs (1 and2) could be streamlined to reduce repetition regarding LMIC burden, poverty, and economic impact.

Response: We have revised accordingly.

**Comment 5:** The manuscript states that cross-country comparative evidence disaggregated by drug resistance status is limited. This claim could be strengthened by stating more explicitly from the outset what evidence already exists/more precise (policy-relevant) evidence gaps (e.g. lack of standardized inequality measures) and what this study might add, better linking the opening paragraph to the points that follow in the introduction. For example: “Previous multi-country studies have reported aggregate catastrophic cost estimates but have not applied standardized inequality measures or systematically disaggregated results by drug resistance status, limiting their usefulness for policy prioritization”.

Response: Thank you. We have a sentence to address this concern. The sentence read as “For instance, while previous multicountry studies conducted by Tanimura et al. [4], Muniyandi [5], Portnoy et al. [6], Akalu et a. [7], and Yan et al. [8] presented aggregated estimates of catastrophic costs, offering valuable insights into the burden of tuberculosis. However, these studies did not employ standardized measures of inequality or systematically disaggregate findings by drug resistance status, limiting their usefulness for policy prioritization.”

**Comment 6:** The definition of catastrophic costs (≥20% of annual household income) is clear, but perhaps the authors could be more explicit about the conceptual link between catastrophic costs and health inequity (eg we know drug resistance status is clinically relevant, but why is it an equity concern?)

Response: We agree that providing a clearer connection between these concepts will strengthen the manuscript. To address this, we have revised the text to explicitly discuss how drug resistance status exacerbates financial burdens and disproportionately affects vulnerable populations, thereby contributing to health inequities. The include text read as “These costs contribute to health inequity, as they disproportionately burden marginalized populations. Notably, drug resistance status is an equity concern because patients with drug-resistant tuberculosis (DR-TB) incur significantly higher treatment costs due to longer therapy and expensive medications. Such costs deepen socioeconomic disparities, as lower-income individuals are less able to afford care, risking poorer outcomes and perpetuating poverty. Thus, analyzing catastrophic costs by drug resistance status is vital for addressing inequities in tuberculosis care and prioritizing vulnerable groups.”

**Comment 7:** It would be helpful for the manuscript to state why HEAT is preferable, and also any potential limitations.

Response: We have addressed this concern in the study strengths and limitation section.

**Comment 8:** Objective are well-formulated, but it may be helpful to clarify the distinction between comparing the magnitude of catastrophic costs (objective 2) and formally measuring inequalities (objective 3). Conceptually, they could potentially overlap.

Response: We appreciate your observation about the potential conceptual overlap between comparing the magnitude of catastrophic costs (Objective 1 and 2) and formally measuring inequalities (Objective 3). To address this concern, we have revised the objectives to clearly distinguish between these three objectives

Methods

**Comment 9:** Please clarify whether the authors have used pre-loaded HEAT data or HEAT Plus (to include data on DR-TB and DS-TB)

Response: We have clarified that the we used preloaded HEAT data that included data on DR-TB and DS-TB. The sentence read as “The study was conducted for 19 LMICs using pre-loaded HEAT data (included data on DR-TB and DS-TB) from national TB Patient Cost Surveys available in the WHO HEAT online software.”

**Comment 10:** The surveys span 2015–2020, but the methods do not explain how differences in survey year were handled. This raises questions about cross-country comparability and should be acknowledged and discussed. It is noted that the discussion section mentions the health systems context and programmatic differences across countries have the potential to affect results and that further research is needed (lines 367-72).

Response: We have addressed this in the study limitation. The sentence read as “Fifth, while our study used a standardized WHO TB Patient Cost Survey tool for data consistency across 19 low- and middle-income countries, surveys were conducted between 2015 and 2020. Minor updates to the tool during this period did not alter core indicators of financial burden, ensuring comparability. However, temporal differences, such as changes in DR-TB treatment regimens or economic conditions, may contribute to observed between-country variations. These factors underscore the need to interpret findings within each country’s specific temporal and contextual framework.”

**Comment 11:** While D, R, PAR, and PAF are defined, it would strengthen the manuscript to briefly justify why these four measures were chosen over others, and how they complement each other in this specific TB context.

Response: We included a paragraph to address this concern. The sentence read as “We selected D, R, PAR, and PAF to assess TB burden and risk factors due to their relevance in epidemiology and applicability to TB. Chosen over alternatives like incidence rates or odds ratios, these measures offer a comprehensive view—D and R address individual impacts, while PAR and PAF inform public health strategies—ensuring a holistic understanding of TB dynamics for effective interventions.”

**Comment 12:** Suggest including in the methods (or in a supplementary file) a description of how each inequality measure is calculated, as well as how the setting average is calculated.

Response: We have added a subsection in the Methods section (or as a supplementary file, if preferred) with the calculation methods and formulas for Difference, Ratio, Population Attributable Risk (PAR), Population Attributable Fraction (PAF), and the setting average. See lines 210-221

**Comment 13:** The study uses pre-loaded TB survey data and automated calculations within the WHO HEAT software. This means the authors rely on data and inequality measures already processed by WHO and calculated automatically by the software. It is unclear which analytical choices were made by the authors (for example, inclusion of countries, handling of subgroups) versus what HEAT did by default. Clarifying this would improve transparency, reproducibility, and reader confidence in the results.

Response: Thank you for your comment on the need for clarity regarding analytical choices versus default processes in the WHO HEAT software. We have revised the Methods section to detail our decisions on country selection, subgroup handling, and use of HEAT’s automated calculations, as provided below, to enhance transparency and reproducibility.

**Comment 14:** The authors note that D and R are unweighted and do not account for subgroup population sizes. It would be helpful to acknowledge this as a limitation and clarify whether it could bias the results, or if the DR-TB and DS-TB groups are similar in size, making unweighted measures appropriate.

Response: We have included a paragraph to address this limitation in the study strengths and limitation section. The sentence read as “Eight, the use of unweighted D and R measures, which do not account for subgroup population sizes could have bias results if subgroups, such as DR-TB and DS-TB, differ significantly in size. In our data from 19 LMICs, DS-TB cases generally outnumber DR-TB cases, potentially skewing unweighted measures toward the larger group. However, as our focus was on relative disparities and this imbalance is consistent across settings, unweighted measures remain suitable for comparison. Future studies could use weighted measures for a more nuanced analysis.”

**Comment 15:** The HEAT software is described, but the exact version, date of access, and any parameter settings used for analysis are not reported. Providing these details improves reproducibility (perhaps as a supplement?).

Response: Since the WHO HEAT software is an online software readily available to the public, we have included the access link in the text. Date of access have been included the reference section.

**Comment 16:** Results provide CI values, but there is no discussion in the methods of how uncertainty is handled/sensitivity analysis

Response: We have include subsection to address the concern. The sentence read as “We reported 95% confidence intervals (CIs) for inequality measures (Difference, Ratio, PAR, PAF) as calculated by WHO HEAT software, accounting for sampling variability in TB survey data per WHO guidelines. Sensitivity analyses beyond HEAT’s outputs were not conducted, as our focus was on standardized measures. However, we checked consistency across subgroup stratifications (e.g., income quintiles, urban/rural) where possible. Limitations due to the lack of extensive sensitivity analysis are noted in the Limitations section.”

**Comment 17:** Sensitivity analysis would be helpful also for key inequality measures, eg, varying default assumptions

Response: Thank you for recommending sensitivity analysis for key inequality measures by varying default assumptions. Due to the lack of disaggregated data in the WHO HEAT database, we were unable to perform such analyses. We have added a statement in the Limitations section to address this constraint, as provided below. The sentence read as “Ninth, due to the absence of disaggregated data in the HEAT database, which provides only pre-calculated outputs, such analyses were not possible. Future studies with detailed data should include sensitivity analyses to assess the robustness of findings on TB financial burden in LMICs.”

**Comment 18:** Line 185: by contextualizing (not contextualize)

Response: Revised.

Results

Overall, results are comprehensive, clearly presented, and appropriately linked to the study objectives.

**Comment 19:** The separation of catastrophic costs from inequality measures makes it easy for the reader to follow

Response: Thank you

**Comment 20:** The inclusion of both country-specific percentages and confidence intervals provides transparency about the precision of estimates. Reporting both absolute (D, PAR) and relative (R, PAF) inequality measures strengthens the interpretation. This also allows the reader to appreciate both the magnitude and the potential impact of reducing inequalities, which is an important contribution to global TB policy discussions.

Response: Thank you.

**Comment 21:** The results clearly demonstrate that DR-TB-affected families generally face higher catastrophic costs than DS-TB families, with Burkina Faso as an informative exception. This directly addresses the study aim of assessing disparities.

Response: Thank you.

**Comment 22:** The text highlights countries with the highest disparities (e.g., Lesotho, Kenya, Papua New Guinea) and provides context for unusual findings (e.g., Burkina Faso), helping the reader understand where interventions may be most needed.

Response: Thank you.

Suggestions / Clarification:

**Comment 23:** The manuscript reports negative values for PAR and PAF and interprets them as indicating potential reduction in catastrophic costs if inequalities are addressed. Because the HEAT software reports PAR and PAF as negative for adverse indicators (where higher values are worse outcomes), it would strengthen the results if the authors explicitly explain that negative values are expected for adverse indicators under the WHO HEAT convention and clarify how these negative values should be interpreted in the context of their study.

Response: Thank you for your valuable feedback on interpreting negative Population Attributable Risk (PAR) and Population Attributable Fraction (PAF) values. We agree that clarifying the WHO HEAT convention for adverse indicators strengthens the manuscript and have added an explanatory statement in the Methods section regarding negative values and their relation to catastrophic costs of tuberculosis. See lines 243-249

**Comment 24:** Table 2: please clarify in caption what the numbers represent (it is not clear in text, as sometimes results described as %, sometimes as magnitude. Eg some ratios (R) are described as “10.4 times reduction” in the text, which seems inconsistent with the table where PAF values are negative percentages. There may be a mismatch between narrative and table metrics that needs clarification. Also please check the -4 value for D in Burkina Faso.

Response: We have revised the text accordingly. The catastrophic costs estimate are reported in percentage while the the inequality measure (D, PAR, and PAF) are reported in percentage points.

**Comment 25:** While CI ranges are provided for catastrophic cost estimates, no CIs are reported for D, R, PAR, or PAF. Including these would allow readers to assess statistical uncertainty in the inequality measures. See comments above in methods-sensitivity analysis, including varying assumptions, should also be described.

Response: Thank you for your feedback on including confidence intervals (CIs) for inequality metrics (D, R, PAR, PAF). We acknowledge their importance for assessing statistical uncertainty. However, the WHO HEAT toolkit data used in our analysis did not provide CIs or disaggregated data to calculate them, preventing this analysis. We have noted this limitation in our study. As suggested, we will expand on sensitivity analyses and varying assumptions in the revised manuscript to enhance robustness. We appreciate your guidance and welcome any further suggestions to address this concern.

**Comment 26:** In figure 1, the caption “Financial inequalities in catastrophic cost incurred by TB-affected families in Lesotho compared to 18 other low- and middle-income countries” could be clearer. It is unclear whether the figure compares all countries or highlights Lesotho specifically. Explicit mention of what the figure shows (absolute vs relative differences, etc.) would improve clarity.

Response: We have revised the title to enhance clarity. The title read as “Figure 1: Financial inequalities in catastrophic cost incurred by TB-affected families in 19 low-and middle-income countries.”

**Comment 27:** While disparities are reported, the results could briefly mention potential factors contributing to the observed differences (e.g., DR-TB treatment duration, additional non-medical costs), even if explored more in the discussion. This would help readers connect numbers to real-world implications.

Response: We have revise the text accordingly.

Discussion

**Comment 28:** The Discussion effectively summarizes key results, including the prevalence of catastrophic costs, differences by DR-TB vs DS-TB, and cross-country disparities. The use of absolute (D, PAR) and relative (R, PAF) measures is appropriately highlighted.

Response: Thank you.

**Comment 29:** The authors reference prior multi-country and single-country studies to situate their findings, demonstrating that the high burden of catastrophic costs among DR-TB households is consistent with global evidence.

Response: Thank you.

**Comment 30:** The Discussion clearly connects findings to actionable implications for TB control policy, UHC, and financial protection interventions (e.g., cash transfers, transport vouchers, decentralization of care).

Response: Thank you.

**Comment 31:** The authors note the cross-sectional design, reliance on self-reported data, country selection, programmatic differences, and the evolving landscape of DR-TB treatment. This transparency strengthens the credibility of the study.

Response: Thank you.

**Comment 32:** The suggestions for longitudinal studies, qualitative research, and intersectional analyses (gender, age, HIV status, rural/urban) are appropriate and forward-looking.

Response: Thank you.

Further limitations/suggestions

**Comment 33:** Lines 257-260: for clarity, I would suggest rephrasing the sentence as follows: “Notably, the PAR and PAF values (reported as negative due to HEAT conventions for adverse indicators) indicate that if the financial burden among DR-TB-affected families were reduced to the level of DS-TB-affected families, the overall prevalence of catastrophic costs could decline considerably.” Also this point seems to be repeated lines 283-5.

Response: Thank you. We have reprhased the text accordingly.

**Comment 34:** It seems the text sometimes uses “times” reduction for PAF, which is inconsistent with HEAT output in percentage form. For example, Lesotho PAF = –10.4% does not mean costs would be reduced 10.4-fold. This should be corrected throughout the Discussion.

Response: Thank you for highlighting the inconsistency in our terminology for PAF in the Discussion section. We have revised the text accordingly.

**Comment 35:** The discussion mentions cross-sectional design and unmeasured confounding, but it could be more explicit that observed disparities cannot be interpreted as causal effects of DR-TB on catastrophic costs, only as associations.

Response: We have revised the text accordingly. The sentence read as “First, the cross-sectional design and unmeasured confounding of the findings shows the observed disparities cannot be interpreted as causal effects of DR-TB on catastrophic costs, only as associations”.

**Comment 36:** The anomaly in Burkina Faso (DR-TB households slightly less affected than DS-TB) is noted, but the Discussion could briefly explore why PAR/PAF are negative there and how this affects interpretation, linking back to HEAT conventions.

Response: We have include a sentence to address this concern, The added text read as “Furthermore, the negative PAR and PAF values observed for Burkina Faso suggest potential reductions in financial burden if inequalities are addressed.”

**Comment 37:** The paper appropriately highlights disparities between drug-susceptible (DS-TB) and drug-resistant TB (DR-TB) households, which is central to the study’s objectives. However, it does not explore additional sub-groups such as age, sex, socioeconomic status, or urban/rural residence. Considering these factors could provide a more nuanced understanding of financial inequalities and identify other vulnerable populations that may benefit from targeted interventions. Though reference to unmeasured confounders lines 280-82, I would suggest a brief discussion or acknowledgment of these potential sub-groups would to strengthen results and ultimately the implications for policy (or perhaps implications for further studies?).

Response: We have addressed this concern in the method section. See lines 182-185. The revised sentence read as “However, we recognize that other dimensions such as socioeconomic status, geographic location, and gender could provide a broader view of disparities, and our focus may not capture all inequalities. Future studies should consider multiple measures for a comprehensive analysis”.

**Other (minor) suggestions**

**Comment 38:** Repetition of statements about DR-TB being a “critical axis of inequality” could be condensed.

Response: We have revised accordingly.

**Comment 39:** Some numerical examples (e.g., D and R in multiple countries) could be summarized more concisely to improve readability.

Response: We have revised the text accordingly. The revise text read as “The difference in catastrophic cost between DR-TB and DS-TB families varies from –4% in Burkina Faso to 74.8% in Lesotho. DR-TB families generally face higher costs, except in Burkina Faso. The largest gaps are in Lesotho (74.8%), Kenya (60%), Papua New Guinea (51%), Uganda (49%), Thailand (47.3%), and Indonesia (43.2%), while the smallest are in Burkina Faso (–4%), Ghana (9%), and Zimbabwe (11%). High costs in Lesotho, Papua New Guinea, Uganda, Thailand, and Indonesia stem from the complexity, duration, and expense of DR-TB treatment. DR-TB families in Lesotho are 5.3 times more likely to incur catastrophic costs compared to DS-TB families, with high ratios also in Kenya (3.3), Thailand (2.6), Papua New Guinea (2.5), Indonesia (2.2), and the Philippines (2.2), while Zimbabwe, Ghana, and Burkina Faso show a ratio of 1.1. These elevated costs for DR-TB are due to costly second-line drugs, extended 18-24 month treatment, and higher risks of treatment failure.”

Overall conclusion and recommendations:

**Comment 40:** Overall, this manuscript provides a timely cross-country overview of inequalities in catastrophic TB-related costs across 19 LMICs and clearly shows that DR-TB-affected households face a greater financial burden. The use of multiple inequality measures and the inclusion of cross-country comparisons are strengths and make the findings relevant for global TB and financial protection discussions.

That said, a few issues should be addressed to improve clarity and strengthen the analysis. In particular, the interpretation of negative PAR and PAF values would benefit from clearer explanation, as these reflect WHO HEAT conventions for adverse indicators and could easily be misinterpreted by readers. While the focus on drug-resistance status is appropriate, it would also be helpful to acknowledge and discuss the potential role of other unmeasured sub-groups (such as age, sex, socioeconomic status, or urban–rural residence) in shaping the observed inequalities. In addition, some form of sensitivity analysis—exploring alternative assumptions within HEAT, such as reference groups, weighting, or catastrophic cost thresholds—would increase confidence in the robustness of the results.

More generally, the paper would be strengthened by going beyond the default HEAT outputs and engaging more explicitly with analytical choices, assumptions, and the underlying cost components driving the observed patterns. Even within the limits of the pre-loaded data, this added depth would improve transparency and enhance the paper’s overall contribution.

Thank you for your thoughtful and constructive feedback on our manuscript. We appreciate your recognition of the timely cross-country overview of inequalities in catastrophic TB-related costs across 19 LMICs and the relevance of our findings for global TB and financial protection discussions. We have carefully addressed most of your comments to improve clarity and strengthen the analysis, as outlined below:

**-Interpretation of Negative PAR and PAF Values:** We have added a detailed explanation in the Methods section to clarify the WHO HEAT convention for reporting negative values for adverse indicators and their interpretation in the context of catastrophic TB costs.

**-Unmeasured Subgroups:** We have acknowledge the importance of other unmeasured subgroups (e.g., age, sex, socioeconomic status, urban-rural residence) in shaping observed inequalities, acknowledging their relevance.

**- Sensitivity Analysis:** We have indicated that nor sensitivity analysis was coinducted beyond the descriptive analysis level in both the method and discussion section.

**-Analytical Choices and Depth:** We have expanded our description with analytical choices, assumptions, and underlying cost components beyond default HEAT outputs to improve transparency and depth.

We hope that we have adequately addressed the reviewers' comments, and we look forward to receiving a favorable outcome on our paper.

Yours Sincerely,

Umaru Sesay

Corresponding Author
